# Highly regulated but can’t be certified as sustainable. Responsible use principles are bridging the gap for biotech trees

**DOI:** 10.1186/1753-6561-5-S7-O53

**Published:** 2011-09-13

**Authors:** Adam Costanza

**Affiliations:** 1 Institute of Forest Biotechnology

## 

Biotech trees are some of the most heavily regulated plants in the world, yet no forest management system certifies the use of them as sustainable. The Responsible Use: Biotech Tree Principles are the first, and only, set of practices designed specifically for the long-term stewardship of biotech trees. These Principles may serve as a framework that certification systems can use when considering whether biotech trees meet their certification standards.

Responsible use principles were developed to guide the long-term stewardship of biotech trees. As forest biotechnology has taken root and genetically modified trees are being field-tested for biofuels, species restoration, and disease resistance, the need for a set of global guiding principles for responsible use of these trees became apparent. Since this need was not being met by the international forestry certification schemes, the Institute of Forest Biotechnology (IFB) developed the Responsible Use: Biotech Tree Principles in a transparent, stakeholder driven process over nearly three years.

A broad set of stakeholders have set aside the issues of whether biotech trees should be used and created these stewardship principles. The Principles are in recognition that responsibly used biotech trees have the potential to benefit society, economies, and the environment in ways that other trees cannot. Central to these Principles are core beliefs that:

• Biotech trees should benefit people, the environment, or both

• Risks and benefits of biotech trees must be assessed

• Transparency is vital and stakeholders must be engaged

• Social equity and indigenous rights are important and must be respected

• Biotech tree use must follow regulations of the appropriate country

Regulations for biotech trees are stringent in most parts of the world. From product inception in the lab, to seedlings grown by tree farmers, it can take years and cost millions of dollars – and the biotech tree may never get deregulated. Long growth cycles make gathering scientific information necessary for deregulation difficult in many countries. However, regulations vary widely in practice from one country to another. We will discuss the regulatory frameworks of biotech trees in Brazil, Chile, U.S., Canada, South Africa, China, and New Zealand.

Certification systems have yet to address biotech tree use in a holistic, scientific manner. We will discuss how the top certification systems in Brazil, Chile, U.S., Canada, South Africa, China, and New Zealand address biotech trees. Along with the regulatory review of these countries, a framework will be developed that shows where regulations and certification systems overlap, diverge, or have significant gaps. This framework will be a tool to begin a dialogue with certification systems on how to best bridge these gaps using the Responsible Use: Biotech Tree Principles.

People have highly disparate opinions about using biotech trees. The reality is that biotech trees are already in use today, and they will be put to use even more in the future. It is important that we engage society, assess environmental risks and benefits, and follow best practices for the stewardship of biotech trees and their products. The IFB, through the Responsible Use Initiative, will work with relevant forest certification systems to provide science-based, stakeholder driven assessments that will help meet the requirements of their own sustainable forestry mechanisms.

The IFB is the only organization to address the sustainability of forest biotechnology on a global scale. The IFB has been in operation for 10 years as a non-profit organization. In December 2010 the IFB published the Responsible Use: Biotech Tree Principles. The Principles along with a comprehensive Biotech Tree Primer are available at http://www.responsibleuse.org. Learn more at IFB’s main website, http://www.forestbiotech.org.

